# Severe persistent pulmonary hypertension of the newborn and dysmorphic features in neonate with a deletion involving *TWIST1* and *PHF14*: a case report

**DOI:** 10.1186/s13256-017-1402-4

**Published:** 2017-08-17

**Authors:** Carina Schinagl, Guro Reinholt Melum, Olaug Kristin Rødningen, Kathrine Bjørgo, Jannicke Hanne Andresen

**Affiliations:** 1 Department of Pediatrics, Oslo University Hospital, Oslo, Norway; 2 Department of Pathology, Oslo University Hospital, Oslo, Norway; 3 Department of Medical Genetics, Oslo University Hospital, Oslo, Norway; 4 Department of Neonatology, Oslo University Hospital, Kirkeveien 166, 0407 Oslo, Norway

**Keywords:** PPHN, Persistent pulmonary hypertension, Craniosynostosis, *TWIST*, *PHF14*

## Abstract

**Background:**

Persistent pulmonary hypertension is a well-known disease of the newborn that in most cases responds well to treatment with nitric oxide and treatment of any underlying causes. Genetic causes of persistent pulmonary hypertension of the newborn are rare. The *TWIST1* gene is involved in morphogenetics, and deletions are known to cause Saethre-Chotzen syndrome. Deletions of *PHF14* have never been reported in neonates, but animal studies have shown a link between severe defects in lung development and deletions of this gene. There have not, to the best of our knowledge, been any publications of a link between the genes *TWIST1* and *PHF14* and persistent pulmonary hypertension of the newborn, making this a novel finding.

**Case presentation:**

We describe a white male neonate born at term to non-consanguineous white parents; he presented with dysmorphic features and a therapy-refractory persistent pulmonary hypertension. Array-based comparative genomic hybridization revealed the presence of a 14.7 Mb interstitial deletion on chromosome 7, encompassing the genes *TWIST1* and *PHF14*.

**Conclusions:**

The *TWIST1* gene can explain our patient’s dysmorphic features. His severe persistent pulmonary hypertension has, however, not been described before in conjunction with the *TWIST1* gene, but could be explained by involvement of *PHF14*, consistent with findings in animal experiments showing lethal respiratory failure with depletion of *PHF14*. These findings are novel and of importance for the clinical management and diagnostic workup of neonates with severe persistent pulmonary hypertension of the newborn and dysmorphic features.

## Background

Persistent pulmonary hypertension of the newborn (PPHN) is a source of considerable mortality and morbidity within the neonatal population. It occurs in approximately 2 per 1000 live born term babies [1]. PPHN describes the failure of the circulatory system to transition after birth, characterized by pulmonary hypertension causing right-to-left shunting and consequently hypoxemia. PPHN is found in neonates with congenital diaphragmatic hernia, meconium aspiration, perinatal asphyxia, or septicemia. Mutations in two genes have been associated with PPHN: *FOXF1* as found in the life-threatening alveolar capillary dysplasia (OMIM 601089) and *TMEM70* (cause of nuclear adenosine triphosphate, ATP, synthase deficiency; OMIM 614052) [2, 3].

The short arm of chromosome 7 contains an important gene for morphogenetics, the *TWIST1* gene on 7p21. This gene is involved in mesodermal cell determination including the ossification process during skull bone formation. Deletions of *TWIST1* cause Saethre-Chotzen syndrome (SCS; OMIM 101400). SCS is characterized by craniosynostosis, maxillary hypoplasia, prominent ear crus, and cutaneous syndactyly [4, 5].

The *PHF14* (plant homeodomain finger protein 14) gene regulates the proliferation of mesenchymal cells through direct involvement in the expression of platelet-derived growth factor receptor α (PDGFRα). PDGFRα up-regulation leads to increased proliferation of mesenchymal cells [6]. Platelet-derived growth factor (PDGF) plays a critical role in lung development, especially of alveolar smooth muscle cells and alveolar septation, but also in the development of smooth muscle cells in arterioles [7]. Lindahl *et al*. showed that PDGFR-/- knockout mice die of respiratory insufficiency due to a lack of development of alveolar muscular layer and alveolar septation [7]. Kitagawa *et al*. showed that an overexpression of PDGFRα leads to increased proliferation of mesenchymal cells with fibrosis and interstitial hyperplasia. In their study *PHF14*–null mice died shortly after birth due to respiratory failure [6]. Huang *et al*. showed severe hypertrophy of the alveolar wall in their study on knockout *PHF14*-/- mice [8]. The alveoli were poorly developed and incompletely inflated, and the mice died of respiratory failure shortly after birth. Heterozygous mice (*PHF14*+/-) presented with similar, but milder pulmonary changes compared to the homozygous mice (*PGF14*-/-) [8].

Deletion of *PHF14* has been described in fetuses with Dandy–Walker malformation [9] and in a human cell-line of biliary tract cancer cells [10]. It has however never been documented in neonates. We are not aware of any prior description of patients with therapy-refractory pulmonary hypertension in conjunction with a deletion of chromosome 7 involving *TWIST1* and *PHF14*, and believe this to be an important differential diagnosis in neonates with this presentation.

## Case presentation

Our patient is a white boy born at 41 + 3 weeks via vaginal delivery; he is the second child of healthy, non-consanguineous white parents. There were no known cases of neonatal deaths or chromosomal or genetic abnormalities in the family. Apart from polyhydramnion detected in week 32, antenatal follow-up was unremarkable. His mother was treated with intravenously administered penicillin prior to delivery due to positive vaginal swab for group B streptococci. Membranes ruptured at delivery, the amniotic fluid was meconium stained. Birth weight, length, and head circumference were 4244 grams (90th centile), 52 cm (50th 75th centile), and 35.5 cm (50th centile), respectively. Apgar score was 9-7-7. Five minutes after birth he appeared cyanotic and did not respond to oxygen. He was intubated at 20 minutes of age and transferred to our Neonatal Intensive Care Unit (NICU) on a ventilator: arterial oxygen saturation (SaO_2_) preductal 80 to 85%, postductal 70 to 75%.

**Fig. 1:**
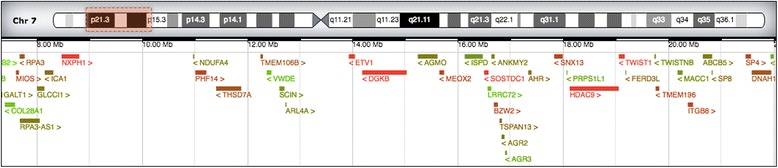
Graphic representation of the deleted region on chromosome 7 (arr[hg19] 7p15.3p21.3(7335077-22073134)x1), including the protein coding genes (from Decipher, https://decipher.sanger.ac.uk/)

Upon examination the following dysmorphic features were observed: hypertelorism, broad and flat nasal bridge with anteverted nostrils, bilateral epicanthus, and asymmetric head shape. His left eye was smaller and positioned lower than his right eye. His hands showed single transverse palmar creases, proximally positioned thumbs, and short pointy fingers bilaterally. His left thumb was also found to be hypoplastic, and there was syndactyly between the fourth and the fifth finger on his left hand. Furthermore we noticed that both big toes were more proximal than normal. A chest X-ray revealed 12 ribs on his right side and 11 on his left side. An echocardiogram revealed a persistent ductus arteriosus with right-to-left shunting and significant pulmonary hypertension. No structural heart defect was seen. A cranial ultrasound found no anomalies. An ultrasound of his abdomen initially showed normal parenchymatous organs in normal anatomical position. Array-based comparative genomic hybridization (array CGH) identified a 14.7 Mb interstitial deletion on chromosome 7 (arr[hg19] 7p15.3p21.3 (7335077-22073134)x1), involving 41 genes, including *TWIST1* and *PHF14* (Fig. 1). The deletion was verified by analyzing G-banded chromosomes (46,XY,del(7)(p15.3p21). Cytogenetic analysis (G-banding) was performed on metaphase chromosomes of our patient derived from peripheral blood lymphocytes cultures according to standard procedures. Deoxyribonucleic acid (DNA) was extracted from whole blood. Array CGH was performed using 4x180K SurePrint G3 Human CGH Microarrays (Agilent Technologies, CA, USA). Labelling and hybridization was performed according to manufacturer’s recommendations. Data were processed using Feature Extraction, and analyzed using Genomic Workbench (Agilent Technologies, CA, USA) with the following settings: algorithm, ADM-2; threshold, 5; minimum number of probes in a region, 4. Genomic positions refer to the Human Genome February 2009 assembly (GRCh37/hg19).

Initial hypotension was treated with dopamine, dobutamine, and epinephrine. Increasing oxygenation problems occurred despite intensive treatment with high frequency oscillations, nitric oxide, sildenafil, and prostacyclin. He developed seizures confirmed by electroencephalogram (EEG), these were treated with phenobarbital. He gradually developed hepatosplenomegaly and elevated liver enzymes. Ultrasound revealed thrombi in the aorta abdominalis and ductus venosus. Treatment with dalteparin was started. Antibiotic treatment was given throughout his stay in NICU, cultures remained negative. Severe therapy-refractory pulmonary hypertension, increasing oxygenation problems, and the genetic findings were reasons to redirect treatment. He died at 14 days of age. His parents agreed to an autopsy.

The autopsy reported a boy with body weight and measurements consistent with gestational age 41 weeks. The lungs appeared immature with dysmorphic alveoli. There was hypertrophy of the pulmonary vasculature and extensive fibrosis of the vessel wall. Large areas of coagulation necrosis were seen in spleen and liver, in addition to mural thrombi in large vessels of the liver, spleen, and lungs. These findings are suggestive of a global activation of the coagulation system. In addition, extensive extramedullary hematopoiesis was seen in the liver, spleen and lungs, considerably more than expected at term. A premature fusion of the skull (craniosynostosis) was seen, affecting both the coronal and sagittal sutures. Dysmorphic features of the face and anomalies of the hands and feet were seen, as previously described. A neuropathological examination revealed hypoxic damage to the brain in the hippocampus and thalamus, at least 1 to 2-weeks old.

## Discussion

In this case report we present a newborn with dysmorphic features and persistent pulmonary hypertension immediately after birth. PPHN is a condition defined by the presence of elevated pulmonary vascular resistance and right-left shunt through the ductus arteriosus and/or foramen ovale. Echocardiography is considered the most reliable noninvasive test to establish the diagnosis. A right-to-left shunt confirms the diagnosis in the absence of a structural heart defect [11].

His initial evaluations demonstrated no structural or anatomic explanation for PPHN, as well as no classic etiologies. Therapeutic management consisting of mechanical ventilation and pulmonary vasodilators did not lead to clinical amelioration. The additional findings of dysmorphic features of face and limbs prompted a genetic workup that showed a deletion on chromosome 7 (7p15.3p21.3). Two genes in this region were brought to our attention: *TWIST1* and *PHF14*. To the best of our knowledge neither of these genes has previously been reported to cause severe pulmonary hypertension. Similar skeletal malformations and dysmorphic features have, however, been described in other case studies encompassing the same deletion [12–14]. Most of these findings are probably caused by haploinsufficiency of *TWIST1* on 7p21.1 (SCS). In our patient both the therapy-refractory PPHN and autopsy results, showing increased musculature and fibrosis of the pulmonary vessel, point to an involvement of *PHF14*. Deletion of this gene has been found to cause lethal respiratory failure in mice, with histopathological examinations showing severe hypertrophy of the alveolar wall and lung fibrosis [6, 8]. *PHF14* is a regulator of mesenchymal growth via regulation of the PDGFRα [6]. Decreased activity of *PHF14* leads to increased expression of PDGFRα, leading to increased proliferation of mesenchymal cells, including fibroblasts, and increased proliferation of smooth muscle cells in the alveoli and the pulmonary arterioles [7]. These processes could explain the findings in our patient. Why there does not appear to be an involvement of *PHF14* in the other cases containing the same deletion remains unknown.

The comparable heterozygote mice in Huang and colleagues’ publication had a much milder phenotype, leading to speculations of involvement of epigenetic factors as explanation of the severe phenotype in our patient [8, 15]. The findings of increased extramedullary hematopoiesis could be explained by increased fibrosis in the medulla, caused by a deletion of *PHF14*. The medulla was, however, not examined and this therefore remains a subject of speculation. As for the state of hypercoagulopathy, we have no good explanation other than the generally poor condition of the patient. Regarding other genes in the region of this deletion, none of these are likely candidates to explain the phenotype and clinical presentation in our patient.

The outcome was considered to be poor due to neurological symptoms and therapy-refractory PPHN. It was therefore decided to redirect treatment.

## Conclusions

Standard evaluation can sometimes fail to detect the cause of PPHN. In cases where extensive treatment of persistent pulmonary hypertension fails to ensure clinical improvement, more extensive evaluations are warranted, such as a genetic workup. In particular, when persistent pulmonary hypertension is coupled with craniosynostosis and dysmorphic features, involvement of *TWIST1* and *PHF14* should be considered. This is, to the best of our knowledge, the first case report of involvement of *PHF14* in a deletion in a neonate.

## Abbreviations

Array CGH: Array-based comparative genomic hybridization
NICU: Neonatal Intensive Care Unit
PDGFRα: Platelet-derived growth factor receptor α
*PHF14*: Plant homeodomain finger protein 14
PPHN: Persistent pulmonary hypertension of the newborn
SCS: Saethre-Chotzen syndrome
